# Successful use of extracorporeal membrane oxygenation for airway‐obstructing lung adenocarcinoma

**DOI:** 10.1111/1759-7714.13623

**Published:** 2020-08-26

**Authors:** Shinsuke Kitazawa, Naohiro Kobayashi, Sho Ueda, Yuki Enomoto, Yoshiaki Inoue, Toshihiro Shiozawa, Ikuo Sekine, Hitomi Kawai, Masayuki Noguchi, Yukio Sato

**Affiliations:** ^1^ Department of General Thoracic Surgery, Faculty of Medicine University of Tsukuba Tsukuba Japan; ^2^ Department of Emergency and Critical Care Medicine, Faculty of Medicine University of Tsukuba Tsukuba Japan; ^3^ Department of Respiratory Medicine, Faculty of Medicine University of Tsukuba Tsukuba Japan; ^4^ Department of Medical Oncology, Faculty of Medicine University of Tsukuba Tsukuba Japan; ^5^ Department of Pathology, Faculty of Medicine University of Tsukuba Tsukuba Japan

**Keywords:** Airway obstruction, extracorporeal membrane oxygenation, lung cancer, oncogenic mutation, targeted therapy

## Abstract

Endobronchial‐invasive lung cancers are generally diagnosed at advanced stages and may require emergency treatment for airway obstruction. Stent implantation is a common intervention for such obstructed airways but certain subsets of patients cannot receive adequate treatment without respiratory support. Veno‐venous extracorporeal membrane oxygenation (ECMO) is a salvage therapy for respiratory failure but its usefulness in managing patients with advanced lung cancer remains unclear given the poor prognosis. In recent years, molecular targeted agents for patients with driver mutations offer rapid responses and may be administered even while under critical care. In this report, we describe the case of 39‐year‐old female who presented to our emergency department with severe respiratory distress. A computed tomography scan revealed a large mediastinal tumor invading the tracheal carina causing severe stenosis of the left main bronchus and right main pulmonary artery. ECMO support was required as the respiratory condition remained unstable despite high pressure ventilation. Under ECMO support, the patient underwent bronchial stent implantation and was successfully weaned off ECMO. The tumor was histologically diagnosed as pulmonary adenocarcinoma with anaplastic lymphoma kinase gene rearrangement. Treatment with a tyrosine kinase inhibitor, alectinib, induced a marked tumor reduction within a short period. The patient recovered well and is now in remission one year later. This case indicates that intensive respiratory support with ECMO may become a bridge through the critical period for selected patients with respiratory failure secondary to advanced lung cancer.

**Key points:**

**Significant findings of this study:**

ECMO was important to maintain oxygenation during airway intervention for acute respiratory failure due to critical lung adenocarcinoma with ALK gene rearrangement.

**What this study adds:**

With the development of targeted therapies and the improvement in therapeutic bronchoscopy, intensive respiratory support with ECMO may be helpful especially in selected lung cancer patients with oncogenic driver mutations.

## Graphical abstract

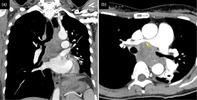

Respiratory support with extracorporeal membrane oxygenation (ECMO) in managing patients with advanced lung cancer remains unclear given the poor prognosis. Targeted agents for patients with driver mutations offer rapid responses and may be administered even while under critical care. In some patients with critical lung cancer, ECMO may become a bridge through the critical period with respect to effective anti‐cancer therapies.

## Introduction

Due to possibly unfavorable prognoses, any indication of extracorporeal membrane oxygenation (ECMO) for patients with advanced lung cancer should be considered individually with respect to risks and benefits.^1^ In general, advanced‐stage malignancy has been thought to be a relative contraindication of ECMO because many cancer‐related, life‐threatening conditions are irreversible.^2^ However, some selected patients can benefit from ECMO support as a bridge between surviving the critical period and starting subsequent anticancer therapy. Here, we report a case of airway‐obstructing ALK‐rearranged lung adenocarcinoma successfully treated by stent implantation and targeted therapy after using ECMO.

## Case report

**Figure 1:**
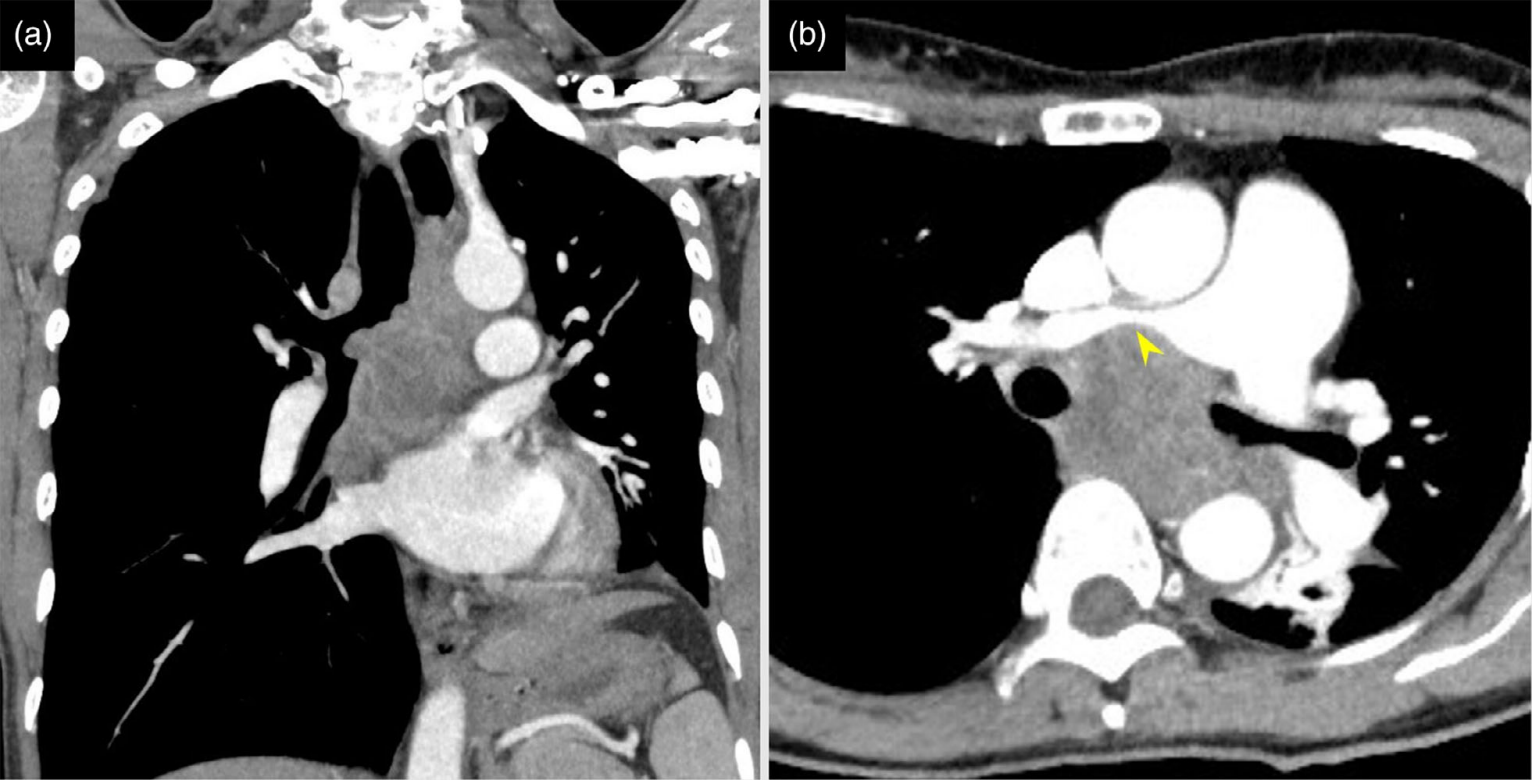
(**a**) Computed tomography revealed a 60 × 48 × 80 mm tumor causing left bronchial obstruction; and (**b**) severe suppression of the right main pulmonary artery (arrow).

**Figure 2:**
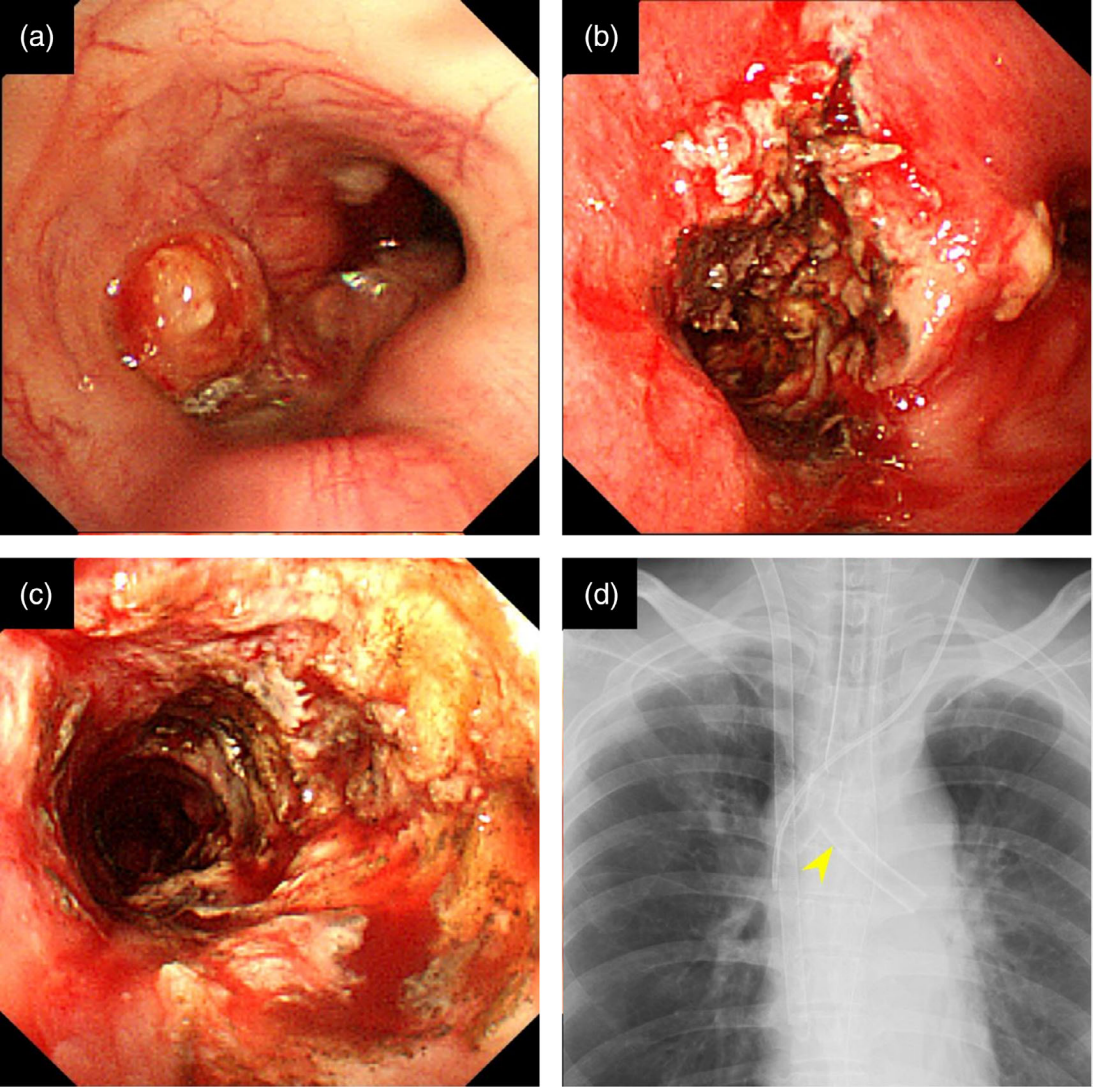
(**a**) Endoscopic examination showing complete occlusion of the left main bronchus; (**b**) the opening of the left main bronchus after tumor reduction by electrocautery; (**c**) the left main bronchus after endoscopic balloon dilation; and (**d**) the stent inserted via rigid bronchoscopy (arrow).

**Figure 3:**
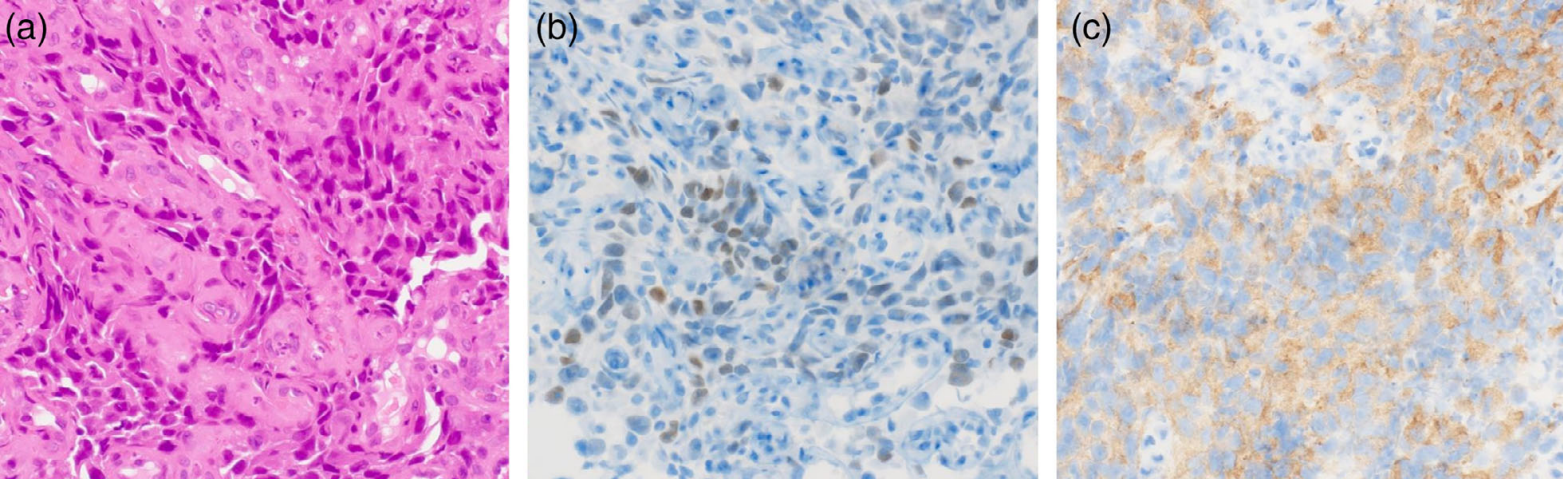
(**a**) A transbronchial biopsy specimen showing poorly differentiated adenocarcinoma; and (**b**,**c**) positive tumor cells for thyroid transcription factor‐1 and anaplastic lymphoma kinase on immunohistochemical staining.

**Figure 4:**
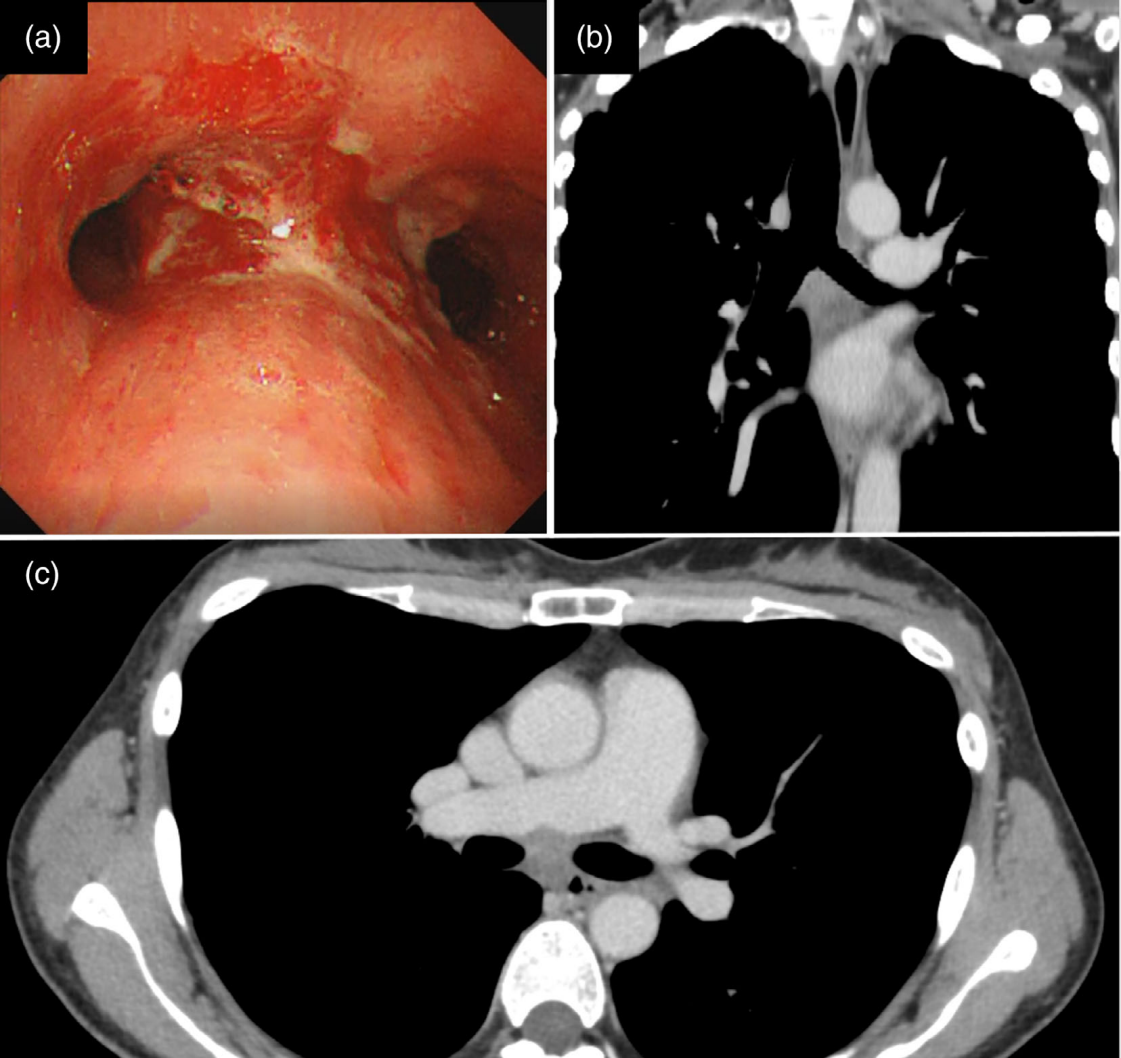
(**a**) Endoscopic examination after three months of alectinib therapy showing opening of the left main bronchus; and (**b**,**c**) computed tomography six months after alectinib therapy demonstrating marked reduction of the tumor.

A 39‐year‐old female with no medical history presented to a referring hospital with complaints of worsening dyspnea. Chest computed tomography (CT) revealed a large tumor primarily in the middle mediastinum invading the tracheal carina (Fig 1a). Furthermore, the left main bronchus and right main pulmonary artery showed severe stenosis from the tumor, restricting pulmonary blood flow to the right lung (Fig 1b). Due to persistent hypoxemia, the patient was intubated and transferred to the ICU of our hospital (day 1). Upon arrival, the patient's condition continued to deteriorate because of the obstructing malignancy. Since high‐pressure ventilation did not improve the patient's respiratory condition, a decision was made to initiate veno‐venous ECMO. Under ECMO support, the patient safely underwent bronchoscopic examination. The tumor was observed to be mainly on the tracheal carina, completely occluding the left main bronchus (Fig 2a). The patient underwent interventional therapy through flexible bronchoscopy using an endoscopic electrocautery device to cut down the tumor (Fig 2b). The stenosis was temporarily relieved after bronchial intervention but the left main bronchus was easily reoccluded due to necrotic tumor tissue. Therefore, stent implantation was deemed necessary to maintain bronchial patency. This procedure was performed on day 11 under general anesthesia via rigid bronchoscopy. The left main bronchus was dilated with a balloon catheter and the trimmed silicon Y‐stent was then inserted (Fig 2c,d). After stent implantation, the patient was successfully weaned off ECMO on day 12 and extubated on day 14. The tumor was histologically diagnosed as poorly differentiated adenocarcinoma (Fig 3a). Immunohistochemical staining revealed that tumor cells were positive for thyroid transcription factor‐1 and diffusely positive for ALK (Fig 3b,c). Moreover, *ALK* gene rearrangement was detected by fluorescence in situ hybridization analysis. The patient received a tyrosine kinase inhibitor (TKI), alectinib, via nasogastric tube from day 16, recovered well and was discharged from the ICU without any adverse events related to the targeted therapy. Six months later, a chest CT scan showed a marked reduction of the tumor (Fig 4a–c). Since the stent had migrated proximally causing slight dyspnea, it was removed under rigid bronchoscopy. The patient remains alive a year after diagnosis without any respiratory symptoms and alectinib treatment is continuing.

## Discussion

According to previous studies, ICU and hospital mortality rates for patients with lung cancer admitted to emergency departments are estimated at 47% and 60%, respectively.^3^ These high mortality rates result from the fact that patients with respiratory failure are unable to receive conventional cytotoxic chemotherapy due to a poor performance status (PS) and high incidence of side effects.^4^, ^5^ In addition, Park and colleagues reported 16 patients with malignant airway obstruction who required ECMO for respiratory failure. They verified the feasibility of stent placement under ECMO support but mean survival time was only four months.^6^ These unsatisfying results raise questions about the futility of intensive respiratory support, including ECMO and mechanical ventilation.

This paradigm has gradually changed with both the development of targeted therapies for lung cancers with oncogenic mutations and the improvement of therapeutic bronchoscopy in the palliative setting of alleviating airway stenosis.^7^ When considering the indication for intensive respiratory support, both the cause of respiratory failure and response to systemic therapy are regarded as crucial factors.^8^, ^9^ Two major clinical conditions will cause respiratory failure in patients with advanced lung cancer. One is diffuse involvement of lung parenchyma due to multiple lung metastases or pulmonary carcinomatous lymphangitis. The other is malignant airway obstruction secondary to airway invasion. In particular, airway obstructions may qualify for intensive respiratory support compared with diffuse parenchymal malignancy because bronchial intervention procedures enable quick recovery from respiratory failure if airway stenosis is relieved.^10^, ^11^ Histological subtype and genetic status of lung cancer are also important factors in predicting the response to chemotherapy.^12^ TKI treatment often results in a rapid and dramatic response that guarantees a better overall survival. In addition, the adverse event profiles of targeted therapies are less than those of conventional chemotherapeutic agents.^13^, ^14^ Thanks to higher efficacy and lower toxicity, targeted therapies can be considered for use in patients with poor PS, even when patients are receiving critical care.^15^ Therefore, there certainly exist some populations with respect to pathology of respiratory failure and oncogenic mutations who would benefit from major intervention during the critical period.

In our case, there are three possible reasons for the successful treatment. First, the patient was a relatively young woman with good organ function and no comorbidities. Second, recovery from respiratory failure occurred as expected when the left main bronchial stenosis was relieved by endobronchial intervention. Third, the patient immediately underwent ALK inhibitors resulting in marked tumor regression within a short period. In contrast, there was also a major limitation regarding this clinical course as the histological diagnosis and mutation status were revealed only after the initiation of ECMO support. This delay in test results may confound treatment decisions as ECMO usage needs to be decided on an urgent basis. Accumulation of clinical experiences will thus be necessary to determine the appropriate indications for ECMO.

In conclusion, here, we describe the successful ECMO support of a patient with malignant airway obstruction who achieved a rapid beneficial response to targeted therapy. Although ECMO support cannot be applied to the majority of lung cancer patients, this report can be a reference to consider the utility of ECMO for advanced malignancy.

## Disclosure

The authors have no conflicts of interest.
